# Quantifying the effect of body mass index, age, and depression severity on 24-h activity patterns in persons with a lifetime history of affective disorders

**DOI:** 10.1186/s12888-016-1023-2

**Published:** 2016-09-09

**Authors:** Nahid Banihashemi, Rébecca Robillard, Jean Yang, Joanne S. Carpenter, Daniel F. Hermens, Sharon L. Naismith, Zoe Terpening, Django White, Elizabeth M. Scott, Ian B. Hickie

**Affiliations:** 1 Charles Perkins Centre, University of Sydney, Camperdown, NSW Australia; 2 School of Mathematics and Statistics, University of Sydney, Camperdown, NSW Australia; 3 Clinical Research Unit, Brain and Mind Centre, University of Sydney, Camperdown, NSW Australia

**Keywords:** Functional linear model, Multiple regression method, Body mass index, Actigraphy, Circadian activity pattern, Depression, Affective disorders

## Abstract

**Background:**

Patients with affective disorders of different ages have been found to present weight changes and different circadian activity patterns. This study assessed the effects of age, Body Mass Index (BMI) and depression severity on the activity-rest cycle in persons with affective disorders using a novel multifactorial 24-h analysis method.

**Methods:**

Two hundred and thirty-six participants aged between 14 and 85 years underwent 5 to 22 days of actigraphy monitoring (mean duration = 14 days). BMI was also recorded and symptom severity was assessed with the Hamilton Depression Rating Scale (HDRS). Participants were divided into two groups: healthy controls (*n* = 68) and participants with a lifetime diagnosis of affective disorders (*n* = 168). First, the multiple regression method was employed to formulate the circadian activity pattern in term of the factors age, BMI and HDRS. For each group, the functional linear analysis method was applied to assess the relative effects of the factors. Finally, Wald-tests were used to assess the contribution of each factor on the circadian activity pattern.

**Results:**

In the affective disorders group, higher BMI was associated with higher activity levels from 3 am until 5.30 am and with lower activity levels from 10 am until 10.30 pm. Older age was associated with less activity across the day, evening, and night - from 11 am until 5.30 am. Higher HDRS scores were associated with higher activity around 1:30 am. In healthy controls, the effects of BMI and age on activity patterns were less pronounced and affected a narrower portion of the 24-h period.

**Conclusion:**

These findings suggest that older age and higher BMI are linked to lower daytime activity levels. Higher BMI and worse symptom severity were also associated with nocturnal activity patterns suggestive of sleep disturbances. The influence of age and BMI on 24-h activity profiles appear to be especially pronounced in people with affective disorders.

## Background

Metabolic changes, such as weight gain, hypertension or diabetes, are highly prevalent in affective disorders. For instance, the rate of metabolic syndrome (characterized by increases in blood pressure, elevated blood sugar levels, abdominal obesity and/or low HDL cholesterol levels) is twice as high in women who experience depression at one stage in their lives compared to women without a history of depression [1], while both men and women with the metabolic syndrome have been found to have a twofold increase in the risk for future depression [2]. Body weight, which is closely related to sedentary lifestyle and sleep abnormalities, is probably one of the most prominent and readily modifiable components of these metabolic complications and there is increasing evidence for related metabolic pathophysiology in obesity and affective disorders [3–5].

Elevated body mass index (BMI) has been reported in both unipolar depression and bipolar disorder [6, 7]. Longitudinal studies have highlighted bidirectional associations between BMI in the obesity range (≥30) and depression, and that BMI in the overweight range (BMI 25–29.99) is predictive of future depression among adults (aged from 20 to over 60), but not younger adults [8]. Importantly, BMI in the overweight range is also predictive of slower response to antidepressants [9, 10], suggesting that weight complications could contribute to less favorable courses of illness. On the other hand, medications commonly used to treat affective disorders may contribute to weight gain [11].

Various aspects of the rest–activity cycle have been associated with BMI. Shorter sleep duration and greater sleep fragmentation have been found in those with greater BMI [12], and stronger associations between sleep duration and BMI have been found in younger age groups [13]. In bipolar disorder, higher BMI is associated with delayed sleep phase [14, 15] and worse sleep disturbances (measured by actigraphy and self-report) [16]. Lower levels of physical activity are also linked to higher BMI in both young and old age groups [17–20]. However, consideration of activity patterns across the entire 24-h cycle is needed to better understand how the activity-rest cycle relates to BMI in people with affective disorders.

The activity-rest parameters are also influenced by affective disorders and associated symptoms across age groups. For example, sleep disturbances, including sleep fragmentation and altered sleep duration, are associated with worse affective symptoms in both young and older adults [21–24], as is reduced daytime physical activity [25–27]. However, given age-related differences in BMI and the activity-rest cycle, relationships between activity patterns and affective disorders are likely to differ as a function of age. Specifically, BMI is well known to increase with age [28]. Furthermore, the activity-rest cycle varies considerably across the lifespan. There is a reduction in overall activity from young to old adulthood, including reductions in both light and moderate to vigorous physical activity, and an increase in sedentary behavior [29, 30]. Increasing age is also accompanied by greater variability and reduced amplitude of the 24-h activity rhythm, reduced sleep time and increased sleep fragmentation [31, 32]. In addition, the timing of rest and activity also changes with age: phase delays occur in adolescence [33] and phase advances occur from young to middle and older adulthood [34]. Importantly, some of these normal age-related changes in the activity-rest cycle have been found to be more pronounced in persons with a history of affective disorders [35]. This could notably be linked to different pathophysiological mechanisms underlying affective disorders in older people as compared to younger people [36–38].

There is a need to evaluate the relative contribution of factors such as age, BMI and depression severity on activity-rest rest-activity patterns in those with affective disorders. The application of Cosinor [39] or non-parametric models [40] on actigraphy data has enabled the quantification of parameters defining circadian rhythms such as the amplitude, strength, and phase of the activity-rest cycle. New techniques are now available to characterize the influence of variables of interest at each time point of the 24-h period. For instance, functional linear modeling (FLM) [41] is a novel method which can define the effects of a given variable on actigraphy time series. FLM is a subset of the Functional Data Analysis method (FDA), an approach gaining increasing attention due to its various applications in biomedical and behavioural research [42]. In the field of FDA, statistical methods are employed to analyze data in non-numeric forms such as images, graphs (e.g., trees), or functions to obtain information from the natural form (e.g., functions, graphs) of the data. The relation between these functions and other variables of interest are formulated via a multiple regression model. Consequently, when this method is applied to actigraphy, the regression coefficients of the model represent associations between these variables of interest and activity across the 24-h period. In addition, this method yields valuable information about the time intervals in which the variables of interest have the greatest influence on activity patterns. This method has previously been used to compare activity data across indices of apnea and BMI in 395 participants presenting to a sleep clinic with suspected sleep apnea, insomnia or restless legs syndrome [43]. Findings showed that individuals with higher BMI had higher activity during the night and lower activity during the day.

The current study aimed to examine 24-h activity patterns in persons with a lifetime history of affective disorders in relation to BMI, age and depression symptom severity using FLM. A novel analysis tool was developed to analyze the relative impact of each variable on activity patterns.

## Methods

### Participants

Participants were recruited from specialized assessment and early intervention services (Youth Mental Clinic [44, 45] and the Healthy Brain Aging Clinic at the Brain & Mind Research Institute, Camperdown and *headspace*, Campbelltown, Sydney, Australia). One hundred and sixty-eight patients with a lifetime history of affective disorders (primary diagnoses: anxiety, *n* = 15; bipolar, *n* = 35; or major depressive disorder, *n* = 118) were included in the current analysis. Their ages ranged from 14 to 83 years and their BMI varied from 15.5 to 49.9. Sixty-eight healthy control participants were included for comparative purposes. Their ages ranged from 18 to 85 years and their BMI ranged from 16 to 34.6. Table 1 reports demographic information.

**Table 1:** Demographic, depression, and BMI characteristics of patients and controls

	Controls	Affective
*n*	68	168
Age (mean(SD))	38.8 (21.4)	35.7 (20.8)
Gender (% female)	59.7	64.7
HDRS (mean(SD))	-	11.5 (7.6)
BMI (mean(SD))	24.8 (4.47)	25.4 (6.0)

*SD* standard deviation; *HDRS* Hamilton Depression Rating Scale; *BMI* Body Mass Index

Lifetime diagnosis of affective disorders was determined by a psychiatrist or trained research psychologist using DSM-IV-TR criteria [46] and current medication information was collected. Medication information was missing for 12 patients (7 %). Of the remaining 156 patients, 63 % were taking at least one medication. The most common medications were SSRIs (21 %), SNRIs (18 %), antipsychotics (18 %), and mood stabilizers/antiepileptics (15 %). None of the control group participants reported any mental disorder. Exclusion criteria for all participants were a history of stroke; neurological disorder; head injury with loss of consciousness greater than 30 min; medical condition known to affect cognition (e.g., cancer, dementia) and other psychiatric illness.

### Procedures

Wrist actigraphy was recorded using actiwatch devices built with equivalent accelerometers and known to generate comparable data (Actiwatch-64/L/2/Spectrum, Philips Respironics, USA [47]). Data monitoring was conducted over five to 22 days (mean = 14 days) with 30 or 60 s epochs depending on the actiwatch model. The Hamilton Depression Rating Scale (HDRS [48]) was administered to all patients by a psychiatrist or trained research psychologist to measure depression symptom severity. Measures of height and weight were taken via direct measurement (*n* = 142) or self-report (*n* = 94) for BMI calculation. Both HDRS and BMI information were collected within three months of actigraphy monitoring.

### Functional Data Analysis (FDA)

To analyze the relationship between activity levels, age, BMI, and HDRS, we employed the FLM. Actigraphy, BMI, age and HDRS data were imported into the R statistical software for analysis. FLM methods were applied based on a new package adapted from the Actigraphy package [43]. We developed additional functions and commands within this package to integrate all parameters of the model. The FDA package in R was also employed for further analysis. FLM considers the functional form of the data which can be obtained through functional smoothing based on the curve fitting method.

### Functional smoothing

Herein, the first process of FDA used functional smoothing to convert discrete activity values measured at each time unit (e.g., 30–60 s epochs) into a function. This function represents the expected activity value at each time point measured. The curve fitting method that we applied on the data for smoothing purpose is the Fourier expansion model. This model was used because of its flexibility in representing the data as a function and its relevance for cyclical/periodic datasets. Let *y*_kj_ be the discrete activity count for patient *k* at time point *t*_*kj*_, then activity was described as the following equation:

$$ {y}_{kj} = {Activity}_k\left({t}_{kj}\right) + {\varepsilon}_k\left({t}_{kj}\right), $$

where *k = 1,2,…,M,* and *M* is the total number of patients and *j = 1, 2,…,T*_*k*_*,* where *T*_*k*_ is the total number of time points for patient *k*. Furthermore, *ε*_*k*_*(t*_*kj*_*)* is the error term at each time point*.* The activity for all participants was averaged for each minute across 24-h. Then the averaged actigraphy data were converted into a functional form by employing a set of basis functions φ_i_(t), i = 1,…,n in which their linear combinations estimate Activity_k_(t_j_) as follows:

1 $$ {Activity}_k\left({t}_{\mathrm{j}}\right) = {\mathrm{a}}_{1\mathrm{k}}{\upvarphi}_1\left({t}_{\mathrm{j}}\right) + {\mathrm{a}}_{2\mathrm{k}}{\upvarphi}_2\left({t}_{\mathrm{j}}\right)+\dots + {\mathrm{a}}_{\mathrm{n}\mathrm{k}}{\upvarphi}_{\mathrm{n}}\left({t}_{\mathrm{j}}\right) $$

where a_ik_ for, i = 1,…,n, are scalar coefficients for patient k. The basis functions can be considered as polynomials (φ_i_ (*t*) = a_1_ + a_2_*t*^2^ + … + a_n_*t*^n^), Fourier basis, (φ_i_ (*t*) = a_1_ + a_2_sin(*t*) + a_3_cos(ω*t*) + a_4_sin(2ω*t*) + a_5_cos(2ω*t*) + … + a_6_cos(nω*t*)), splines and wavelets. Note that in Eq. (1), n is the maximum number of basis functions. We have applied different basis functions which give the same results. We used *n* = 21 Fourier basis functions to estimate the patient's activity level in Eq. 1 which captures the major trend with reduced noise. The Fourier basis functions are as follows:

$$ {\upvarphi}_1(t) = 1,\ {\upvarphi}_2(t) = \cos (t),\ {\upvarphi}_3(t)= \sin (t), \dots,\ {\upvarphi}_{20}(t) = \cos \left(21\omega t\right),\ {\upvarphi}_{21}(t)= \sin \left(21\omega t\right), $$

where T is the period, in our case T = 1440 (number of minutes in 24 h) and ω = 2π/T. The coefficients a_ik_, k = 1,…,n of Eq. 1 are estimated by the least squares technique [49]. Finally, a single 24-h functional circadian activity pattern for each participant is obtained by applying the Fourier basis functions that can be used to estimate patient's activity level at any time point throughout the day.

### Functional linear models and multiple regressions

FLM can provide valuable insights about differences between activity patterns across different subgroups. The relation between the circadian activity pattern, age, BMI, and HDRS was formulated as a functional multiple regression model as follows:

2 $$ Activity(t) = {\upbeta}_0(t) + {\upbeta}_1(t)\ \mathrm{B}\mathrm{M}\mathrm{I} + {\upbeta}_2(t)\ \mathrm{Age} + {\upbeta}_3(t)\ \mathrm{HDRS} + \varepsilon (t), $$

where the (t) notation indicates functions over the circadian period for activity (fitted by the Fourier expansion to the actigraphy data for each subject), Activity(*t*), the circadian activity pattern, β_1_(*t*), β_2_(*t*) and β_3_(*t*) are the functional regression coefficients indicating how the circadian activity patterns changes for different affective disorder subjects with different BMI, age and HDRS, ε(*t*) is the functional error term. Also, β_0_(*t*) is a constant term at time t. Note that BMI, age and HDRS are used as continuous variables (predictors) here which is considered as one of the advantages of FLM. As in the multiple regression models, we are interested in estimating regression coefficients in Eq. (2) that produce the group-specific mean circadian activity patterns, and test the relative contribution of these coefficients for circadian activity patterns in each group. Equation (2) is similarly employed for formulating the effect of BMI and age on the activity patterns of healthy control subjects in which the HDRS factor is assumed to be zero. In order to obtain the differences between these activity patterns in term of all covariates, we applied F-tests. As a result, we estimated the time intervals in which the differences are significant. We set a global and point-wise test of significance at a level of 0.05.

### Wald-tests of regression coefficients

In order to evaluate the significance of each variable (BMI, age, and HDRS) in the regression, Wald-tests were employed. This test examines the probability of obtaining zero value for each regression coefficient β_1_ (t), β_2_ (t), and β_3_(*t*). This test for β_1_ (t) results in checking the following null hypothesis:

$$ {\mathrm{H}}_0:\ {\beta}_1(t)=0, $$

against the alternative:

$$ {\mathrm{H}}_{\mathrm{a}}:\ {\beta}_1(t)\ne 0. $$

For β_1_(*t*), the t-value is defined as follows:

$$ \mathrm{t} = {\upbeta}_1(t)/\mathrm{Standard}-\mathrm{error}-\mathrm{of} - {\upbeta}_1(t) $$

In order to reject the null hypothesis, 2.5 and 97.5 % percentile of t distribution (df = 167 for affective and 67 for controls) are used as critical values. If the null hypothesis is rejected the regression coefficient at that time does have an effect on the activity value. Note that the t-value of the regression coefficient represents the contribution of its covariates (BMI, Age or Depression severity) to the activity value independently of the others.

## Results

**Fig. 1:**
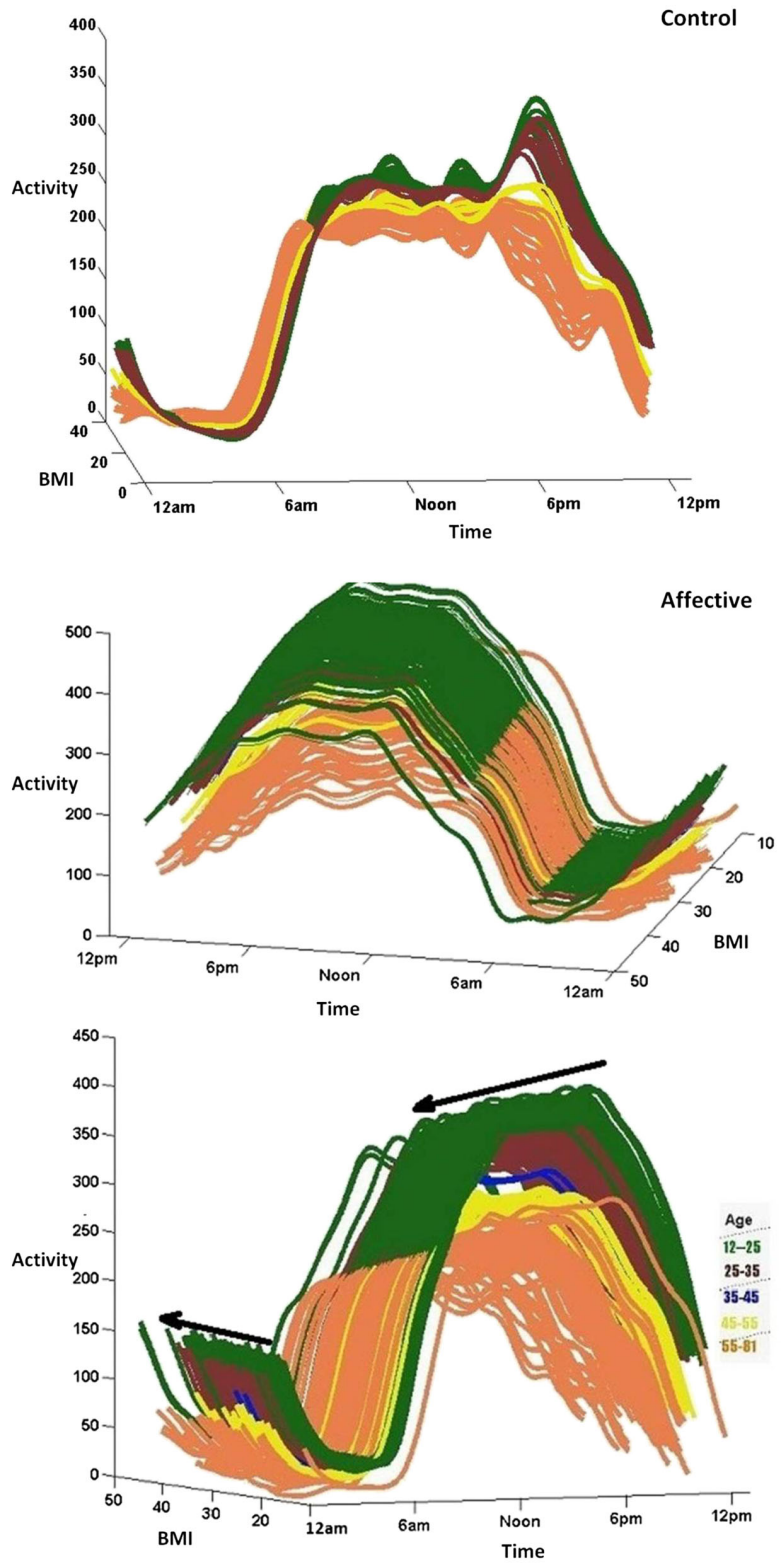
Circadian activity patterns over 24-h according to body mass index (BMI) and age. Different colors represent different age groups in order to illustrate the differences in activity patterns, but this categorization was not applied in the analysis (i.e. all parameters are considered as continuous variables). Top panel: healthy controls, Lower panel: participants with a history of affective disorders. The black arrows on top of the curves show the reduction or increase of the activity values by growth of BMI values

For presentation purposes, best-fit curves for activity patterns over the 24-h period in relation to BMI and age are shown in Fig. 1. From this Figure, it can be observed that participants from the affective disorders group between 12 and 35 years of age have similar activity patterns across the 24-h period, and that the overall activity levels appear to decrease with advancing age. Furthermore, participants from the affective disorders group with higher BMI are somewhat less active during the daytime and more active during the nighttime.

**Fig. 2:**
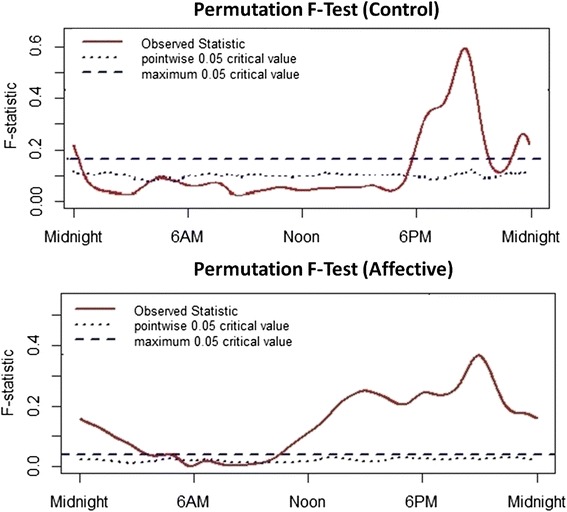
Results from the permutation F-test to obtain the time intervals at which the regression model significantly predicts activity levels. Top panel: healthy controls, Lower panel: participants with a history of affective disorders

Figure 2 shows the F-test results indicating whether the global model integrating BMI, age and HDRS was significantly predictive of activity levels. For the control group, the model reached statistical significance from about 5.30 pm until half-past midnight and again from about 5 am to 5.30 am. For the affective disorders group, the model reached statistical significance between approximately 10 am and 5.30 am.

**Fig. 3:**
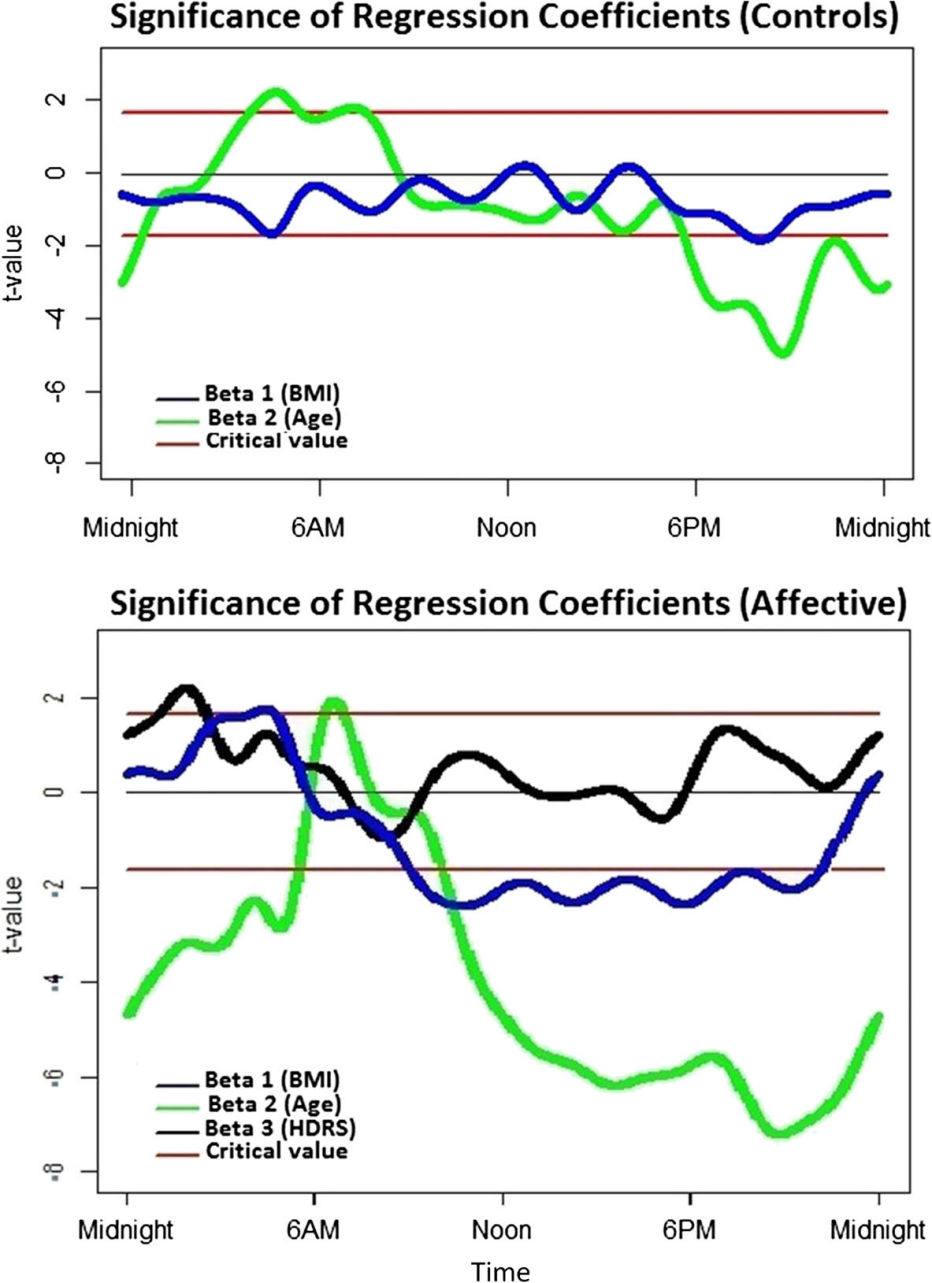
Values of the Wald-test on the regression coefficients for BMI (body mass index), age and HDRS (Hamilton Depression Rating Scale) in the multiple regression model. Red lines represent critical t-values (two-tailed: 0.10). Top panel: healthy controls, Lower panel: participants with a history of affective disorders

**Fig. 4:**
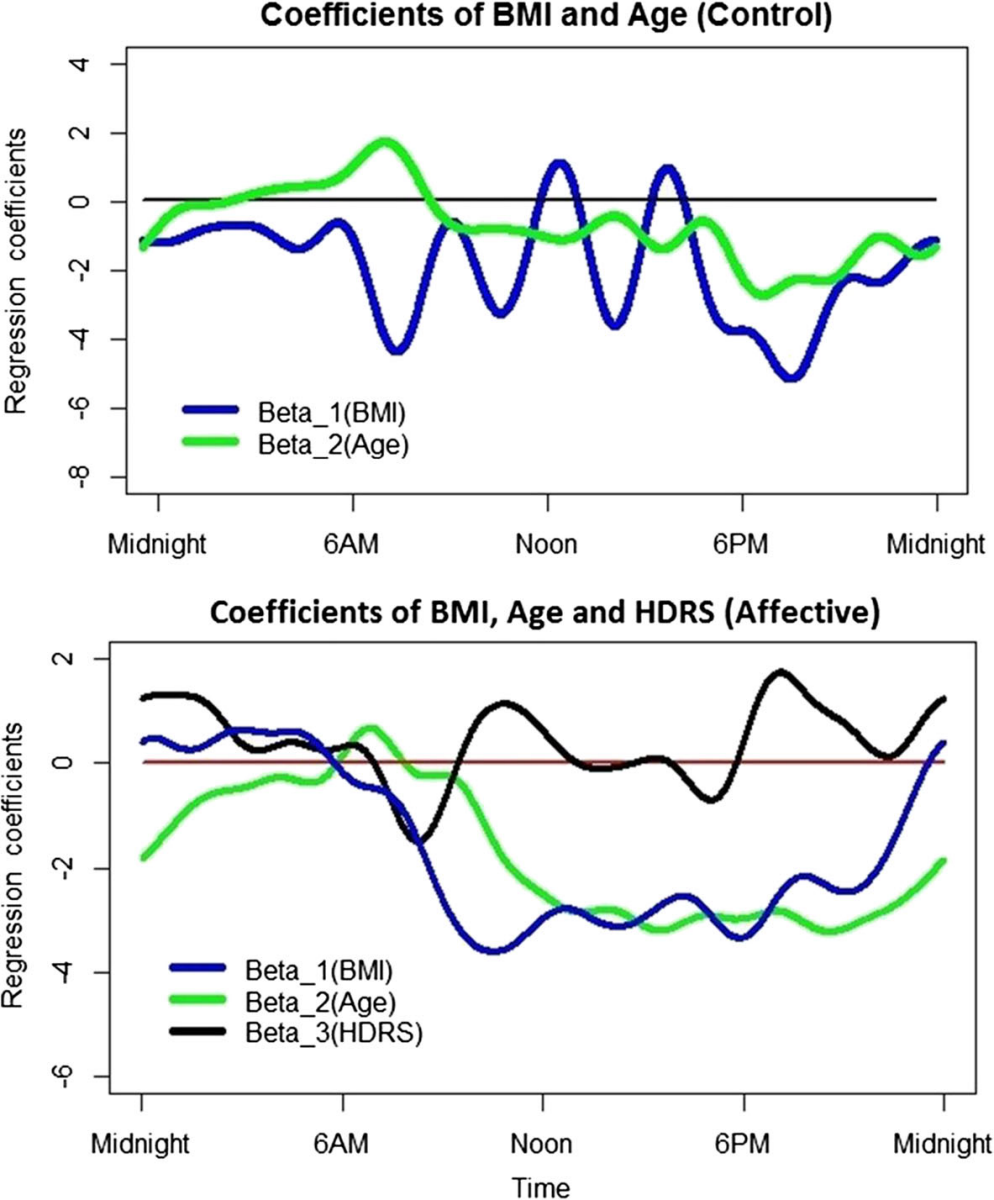
Coefficients of BMI (body mass index; β1(t)), age (β2(t)) and HDRS (Hamilton Depression Rating Scale; β3(t)) in the multiple regression models for controls (top graph) and participants with a history of affective disorders (bottom graph). Top panel: healthy controls, Lower panel: participants with a history of affective disorders

After completing functional smoothing using the 21 Fourier basis functions, the regression coefficients β_1_(*t*), β_2_(*t*) and β_3_(*t*) were obtained to reflect the effects on activity patterns of BMI, age, and HDRS respectively. Wald-test t values for the BMI, age and HDRS regression coefficients (determining whether each covariate brought a significant contribution to the multiple regression model) are represented in Fig. 3. Figure 4, presents the β_1_(*t*), β_2_(*t*) and β_3_(*t*) coefficients which reflect the magnitude and direction of the relative contribution of each BMI, age and HDRS.

In the control group, between 5.30 pm until half-past midnight (when F-test results were significant), lower activity levels were significantly associated with older age and tended to be associated with higher BMI. The influence of BMI reached statistical significance from about 7 pm to 8.30 pm. Furthermore, between 5 am to 5.30 am (i.e. another period where the global model’s F-tests were significant), higher activity was associated with older age and lower BMI.

In the affective disorders group, the regression coefficients reached significance on several occasions within the time period where the model was found to be significant (i.e. between 10 am and 5.30 am). Higher BMI was significantly associated with higher activity levels between 3 am and 5.30 am and with lower activity levels between approximately 10 am and 10.30 pm. Older age was associated with lower activity levels from 11 am until 5.30 am. Higher depression severity as measured by the HDRS was associated with higher activity levels around 1.30 am.

## Discussion

In this study, the impact of BMI, age, and depression symptom severity on the activity patterns of 168 persons with a lifetime history of affective disorders and 68 healthy control participants was analyzed using an adapted FLM technique allowing the examination of multiple factors across all the 24-h cycle. The results suggest that BMI and age influenced activity patterns more broadly across the 24-h period in the affective disorders group than in the control group. In the control group, older age was associated with lower activity levels during the evening and higher activity levels for a brief period in the morning. Conversely, in people with affective disorders, age had a more widespread impact on daytime and nighttime activity patterns, with higher activity in those with younger ages at all times except in the morning. This is in line with the previously reported shift to earlier sleep-wake schedule and the overall reduction in activity with increasing age [29, 30, 32], but suggests that depression may further extend age-related decreases in activity levels*.* Accordingly, previous findings have demonstrated altered activity patterns in people with a history of affective disorders from different age groups, with younger individuals showing delayed sleep and activity schedules in comparison to older people [35]. Furthermore, our findings are in line with the results of Boudebesse et al. [16] which found that higher BMI correlated with worse sleep disturbances in people with bipolar disorder.

Within our sample of persons with a lifetime history affective disorders, depression severity was associated with higher activity levels in the first portion of the night. This could notably be related to delayed sleep onset in a subset of these participants with more severe depression.

BMI also had a significant influence on activity patterns, especially in those with a lifetime history of affective disorders. Higher BMI was associated with lower activity levels across the day and higher activity levels in the later portion of the night. Similarly, previous research highlighted that higher BMI is linked to lower activity levels during the day time in healthy non-depressed individuals [17–20], and to a combination of higher nocturnal activity and lower daytime activity in adults with suspected sleep disorders [43]. This profile is suggestive of sedentary lifestyle and sleep disturbances, two factors known to relate to weight gain.

Although the current findings do not yield information about causality, one could hypothesize that the sedentary lifestyle and poor sleep often resulting from affective disorders could contribute to weight gain and higher BMI. Elevated BMI is also commonly associated with sleep disordered breathing [50], a condition classically leading to multiple brief arousals during the night. In some individuals with affective disorders, it is therefore possible that weight gain may be accompanied by more frequent occurrences of abnormal respiratory events resulting in more pronounced sleep fragmentation. Interestingly, we observed increased nocturnal activity mostly during the later portion of the night, a period rich in REM sleep during which respiratory deficiencies are more likely to occur. Since sleep disordered breathing is also a risk factor for depression [51], it could potentially preclude and/or enhance mood disturbances in some individuals.

Although the observed modulation of activity by BMI and age was largely independent of current depressive symptoms within individuals with affective disorders, the presence or absence of an affective disorder seemed to have a considerable impact. This could suggest that some factors specific to the presence of an affective disorder may potentiate the interactions between BMI and the 24-h patterns of activity. For instance, previous findings suggest that some subtypes of affective disorders are associated with slower metabolic rate [52] and that recovery from major depression is accompanied by an increase in resting metabolic rate [53]. Furthermore, antidepressant medications have been shown to slow down resting metabolic rate in a small sample of individuals with depression [54]. Importantly, in the context of low physical activity levels, global metabolism relies more heavily on resting metabolic rate. Consequently, the impact of sedentary lifestyle on weight gain may be more pronounced in depressed individuals, especially those who take antidepressants. Since higher BMI is in turn linked to higher risk of depression and slower treatment response, it may be relevant to investigate whether the effects of activity patterns on BMI relate to the course of affective disorders, notably in terms of chronicity and relapse.

The present study is limited by its cross-sectional design which does not allow conclusions to be drawn regarding causality. In addition, while our sample of participants ranged between 14 and 85 years of age, it contained few middle age individuals in comparison to older and younger participants. The mean age of the control group was higher than that of affective disorders group. In this regard, a recent meta-analysis suggested that the degree of age-matching across patient and control groups influences group differences in sleep duration [55]. However, the present study did not aim to directly compare participants with and without affective disorders, but rather to investigate how age, BMI and depression severity (as continuous variables) predict changes in 24-h activity patterns within each one of these two clinical groups separately. Also, since our models were designed to report the relative contribution of age and other variables of interests, the effects reported for these other variables are deemed to be independent from the effects of age. Most participants were taking psychotropic medications, many of which are known to influence weight and sleep, although this is broadly representative of the clinical population. Furthermore, some of the BMI values were generated from self-reported height and weight rather than objective measurement. Also, the range of BMI was somewhat smaller in the control group than in the affective disorders group.

## Conclusion

Based on the analysis of multiple days of actigraphy using a novel multifactorial technique, the present study indicates that BMI and age have a considerable impact on 24-h activity patterns. This appeared to be more marked and widespread across the 24-h period in people with a lifetime history of affective disorders than in healthy controls. These findings suggest that, in the context of affective disorders, older individuals and those with higher BMI appear to be prone to more sedentary lifestyle, while those with higher BMI and worse symptom severity also present an activity profile suggestive of restless or delayed sleep. This underscores the importance of dietary, exercise, sleep and metabolic factors in the treatment of psychiatric disorders.

## Abbreviations

BMI: Body mass index
FDA: Functional data analysis
FLM: Functional linear modeling
HDRS: Hamilton depression rating scale
